# Digital Management of Solar Energy En Route to Energy Self‐Sufficiency

**DOI:** 10.1002/gch2.201800105

**Published:** 2019-04-07

**Authors:** Mario Pagliaro, Francesco Meneguzzo

**Affiliations:** ^1^ Istituto per lo Studio dei Materiali Nanostrutturati CNR via U. La Malfa 153 90146 Palermo Italy; ^2^ Istituto di Biometeorologia CNR via G. Caproni 8 50145 Firenze Italy

**Keywords:** distributed generation, energy self‐sufficiency, Internet of Energy, solar energy, solar hydrogen

## Abstract

The Internet of Things approach to manage clean electricity produced from sun, wind, and water is, alongside the hydrogen fuel cell and the Li‐ion battery, the key enabling technology for the transition to 100% renewable energy. Practical examples from distant countries are used to show how today's digital control technology is actually used to ease the generation and utilization of clean, reliable, and cheap solar energy on an annual basis. The conclusions are of relevance also to the redesign of energy education programs in response to dramatic changes in energy technologies.

## 1 Introduction

The ongoing global uptake of renewable energy through large‐scale adoption of solar photovoltaic (PV)^1^ and wind^2^ power is so significant that the global shift to emission‐free, 100% renewable electricity generation is becoming feasible.^3^

The huge and rapid increase of intermittent renewable power generators connected to the grid has led transmission service operators to install at power plants, distribution centers, and “prosumer” (i.e., a consumer who is also a producer) premises various information and communication technology devices for the monitoring, analysis, and control of the grid so as to solve the reliability problems posed by bidirectional energy flows.^4^

Similarly, “Internet of Things” technologies such as smart meters relying on bidirectional communications with local gateways and remote control stations are embedded in PV modules and in wind turbines to optimize renewable energy generation.^5^

The connectivity, automation, and tracking of such sensors, actuators, and smart meters, furthermore, generate a large amount of data suitable for “big data” analysis and processing.^4^

In the scientific literature, the term “Internet of Energy” is defined in two different notions: i) using the Internet parallelly to the grid for enhancing stability, and ii) operating distribution grids as the Internet,, that is, in an energy packet approach. Numerous recent scientific articles^5^ and books^6^ describe the Internet of Energy approach supporting the power infrastructure in which prosumers buy and sell energy back to the grid.^7^

This study focuses on a complementary approach, namely how concepts borrowed from the “Internet of Things” architecture actually assist in clean and renewable energy uptake and utilization on the path to an evolved distributed generation scenario in which clean energy producers become autonomous from the grid, self‐generating their electricity and heat needs from renewable energy sources, particularly sunlight and wind.

Following a practical perspective outlining the use of digital technology since the early days of photovoltaic energy utilization, we use recent examples from countries and regions as distant as Australia, Sicily, Sweden, and Thailand to show how today's “smart” digital control technology is actually being used to ease the utilization of intermittent solar energy on a 24 h basis.

Being of general value, the conclusions are of relevance to the redesign of energy education programs in response to changes in energy technologies.

## 2 A Practical Perspective

Mostly aimed at policy makers, several recent energy scenarios targeting country‐level and even global 100% renewable energy by 2050 address with varying degrees of realism how the transition should be accomplished.^3^

These scenarios help to understand the grand scope of the efforts required, including largely neglected social aspects since energy systems are “socio‐technological systems that involve not only machines, pipes, mines, refineries, and devices but also the humans who design and make technologies, develop and manage routines, and use and consume energy.”^8^

On the other hand, citizens and company's managers need to get a clearer understanding of what is *already* possible by merging renewable energy distributed generation, energy storage, and “Internet of Things” technologies.

Today's PV, reliable, low cost, and efficient generation technology has since long reached the generation parity with the cheapest fossil energy source (coal) in many countries. The main issue that hindered the transition to 100% renewable energy was the lack of affordable energy storage technologies, needed to fix the inherent issue represented by the variability and intermittency of sunlight and wind across any time scale.

This obsolescence is finally being addressed. Solar hydrogen converted with air in today's fuel cells and the Li‐ion battery have emerged as the best technology options to store clean electricity and make both energy self‐sufficient buildings and electric vehicles a viable reality.^9^

Indeed, today's off‐grid solar systems comprised of reliable and lightweight Li‐ion batteries and solar PV modules are finally bringing electricity to vast regions of Africa, India, Bangladesh, Pakistan, Afghanistan, Myanmar, Latin America, Nepal, Vietnam, and other countries where the grid is simply absent. Starting from solar powered irrigation,^10^ photovoltaic electricity made continuous via storage enables indoor and outdoor lighting, mobile phone recharging, connection to the Internet, access to radio and television, and so on, making a major impact on the living and working conditions of entire villages.^11^

None of these applications would have been possible without the discovery, in 1984, of the “power optimizer,” namely the maximum peak power tracker (MPPT) digital electronic technology invented by Atkinson while studying at the University of Queensland and shortly afterward commercialized by the company he founded in Australia.^12^

The MPPT is a digital control technology nowadays present in all the inverters (DC–AC converters), but also in all solar chargers (DC–DC converters), connected and controlling the PV array, including single PV modules used in LED lighting systems. In practice, the MPPT actively measures the voltage and current of the PV array (and therefore the power) and through an iterative and corrective process by posing each time the optimum load, arrives at the maximum power point. This allows to literally “extract” from the PV array the maximum amount of power possible.^13^

The amount of sunlight, temperature of the solar cells, and even their different aging degree constantly change. In addition, for a given level of light intensity (insolation), the solar cells have a characteristic power output curve which relates the actual output power of the cell array to the resistance of the electrical load applied to them.

Prior to the invention of the MPPT, the old DC–AC and DC–DC converters used with photovoltaic cells were unable to maximize the power delivery at any time, as they were unable to change their operating parameters (i.e., voltage and current levels) to ensure that the photovoltaic cells operate at their maximum power point.

**Figure 1:**
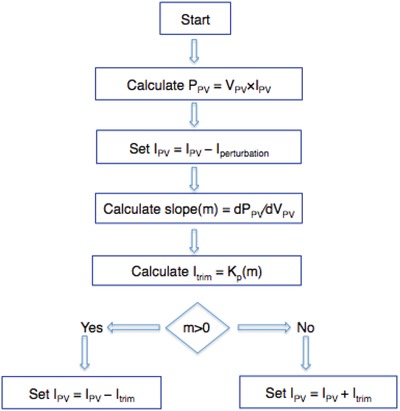
A simplified algorithm used by a typical MPPT, based on Ref. [12].

**Figure** **1** shows a typical algorithm used by the electronic tracker.^13^ The MPPT calculates its location on the power curve slope by changing the current by a small amount, *I*_perturbation_. The variation leads either to a positive or to negative slope in power output, so that the next perturbation is decreased or increased until the slope becomes zero, which is the maximum power point, corresponding to the maximum power extractable from the solar PV module. The value of the iterative current *I*_trim_ is varied proportionally to the magnitude of the slope to allow the system to quickly approach the MPP point.^13^

Another major progress occurred in 2006 when an Israeli company commercialized the first inverter combining the advantages of lower cost single inverter with the possibility to optimize power in single modules thanks to microinverters.

With conventional inverters, the low performance of a single PV module due for example to shading, soiling, or even uneven module aging shading, results in power losses with the overall result of reducing the energy performance of all modules in the string.

**Figure 2:**
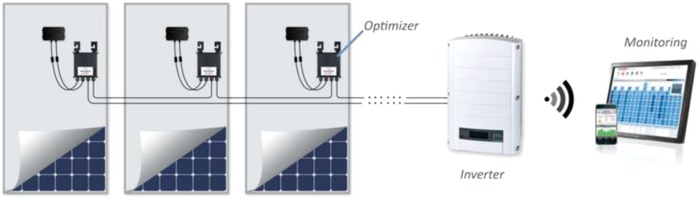
Using power optimizers integrated into each panel, shaded panels do not bring down the entire string performance due to panel mismatch. (Image courtesy of Solar‐Edge).

Now, single DC to DC power conversion units (power optimizers) integrated in all solar modules comprising the PV array work together to deliver the optimum string voltage to the single inverter maximizing the power extractable from a single PV module. The power optimizers connect to each solar module (**Figure** **2**) enabling them to perform independently, providing greater energy production, enhanced safety, and constant information from each panel.^14^

Besides affording up to 25% more energy harvested from the PV system in the course of the year, this approach opens up new possibilities including the possibility to replace old or faulty modules on systems with new, more powerful modules, or insert modules with different orientation or tilt in the same string, as the optimizers smoothly function with multiple module sizes and models on the same string with each panel operating at its maximum power point. Furthermore, lowering the installer's costs, longer string lengths of different lengths are now possible with no need for matching string lengths.

The optimizers offer a range of benefits including panel level monitoring to provide real‐time power generation data from every panel in a solar array, so that, for example, underperforming panels can be highlighted quickly. Finally, the optimizers allow to considerably enhance the overall safety of PV systems as they are designed to automatically reduce the output voltage of each panel to 1 V during installation or when the grid or inverter is shut down (including during maintenance) keeping the overall string and array voltage below risk levels.

Similarly, using a low cost micro‐controller of today, driven by properly developed energy management software, solar‐powered LED luminaires become capable of self‐adapting to the solar environment where they are installed, and predictively manage their output to ensure each light performs optimally in each location thanks to a self‐learning controller which optimizes lighting performance in different conditions.

On August 27–28, 2017, the city of Houston in Texas was hit by hurricane “Harvey” which largely flooded the city including the 250‐acre Gene Green Park. The 156 LED solar light fixtures lighting the park continued to shine with no interruption demonstrating how robust today's off‐grid solar technologies are when compared to conventional luminaires connected to the grid.^15^

Solar street lighting is a key sustainable technology currently solving light poverty in several regions of the world in Africa and Asia as well as in many other regions where the economic costs of conventional street lighting are no longer sustainable.^16^

## 3 Cheap, Clean, and Reliable

In the renewable energy scenario of tomorrow, the volatile demand of energy in many countries, that is currently met by the combination of energies produced from sun, wind, and water (renewable energy sources, RES) plus that from fossil energy sources (FES, Equation (1)), will be met by renewable energy only (Equation (2)):^17^

(1) $$\underset{\left(Volatile\right)}{\mathrm{Demand}}=\underset{\left(Volatile\right)}{\mathrm{Production}\;\mathrm{from}\;\mathrm{RES}}\;+\underset{\left(Plannable\right)}{\mathrm{Production}\;\mathrm{from}\;\mathrm{FES}}$$

(2) $$\underset{\left(Volatile\right)}{\mathrm{Demand}}=\underset{\left(Volatile\right)}{\mathrm{Production}\;\mathrm{from}\;\mathrm{RES}}$$

Remarkable recent progress in weather forecasting, particularly through neural network algorithms leveraging on historical meteorological data, enables predictions of renewable energy production from sun and wind of ever increasing accuracy.^18^

This allows to maximize the hourly and daily input of renewable energy into the grid making renewable energy predictable, even though intermittent, and electricity supply ever more reliable, ultimately solving what Mauro, a scholar in artificial intelligence, has called the “new energy trilemma,” meaning that we could not have “energy that is at the same time cheap, clean, and reliable.”^17^

To maximize revenues and ensure better estimate of the return on their investment, many utilities and investor owners of large PV and wind energy parks today purchase similar energy forecasting services from companies specializing in weather‐energy forecasting.^18^

Similarly, “machine learning” algorithms, that is, algorithms through which a computer learns from past experience and tries to capture the best possible knowledge to make accurate decisions, currently help achieve longer service intervals for natural gas turbines of a large thermoelectric gas turbine manufacturer (algorithms automatically analyze operating data, environmental conditions, and component properties and suggest the most suited operating conditions to prolong the life of the steel turbines).^19^

Carrying ever increasing amounts of renewable electricity, the grid will continue to fulfill the power needs of large manufacturing plants. At the same time, aiming for clean energy self‐sufficiency, companies, families, and individuals will increasingly disconnect from the grid to become users of renewable electricity self‐generated from sunlight (with the PV technology) and from wind (via wind turbines).

Far from being an unrealistic goal, this shift is now possible largely thanks to concomitant progress in photovoltaic generation *and* electricity storage in Li‐ion batteries, as well as solar hydrogen produced via water electrolysis and then used along with air to generate electricity and heat in today's compact fuel cells.

The following case studies from distant countries and sectors demonstrate the dramatic potential of digital technologies transforming objects into agents as well as of new and energy storage solutions to enhance energy self‐sufficiency based on distributed generation and proper management of the clean self‐produced energy.

### 3.1 A Self‐Sufficient Plantation in Sicily

**Figure 3:**
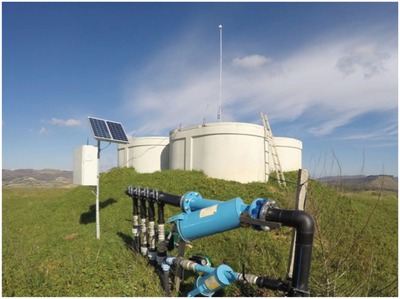
Water tanks serving part of the irrigation needs of an *Opuntia ficus‐indica* plantation in Sicily, Italy. (Image courtesy of Agrowireless).

An *Opuntia ficus‐indica* plantation in Sicily now uses a system of sensors and actuators powered by PV power only (**Figure** **3**) through which all main agriculturally relevant properties are monitored and acted upon. A simple wireless system transmits all data using radio frequency including the values of the pressure in the waterworks, the level of water in the water tanks, air temperature and ground humidity to optimize irrigation, to a “cloud” server.

The plantation has a large surface area (close to 100 ha) with poor and often absent GSM (Global System for Mobile communication) signal. Radio modules using a wireless mesh network made up of radio nodes organized in a mesh topology in which the single modules autonomously choose the shortest path eventually reinforcing the communication signal.^20^

Any command sent, for example, by the plantation manager through his smartphone, is converted into a radio signal and then sent through the mesh network to the destination module.

**Figure 4:**
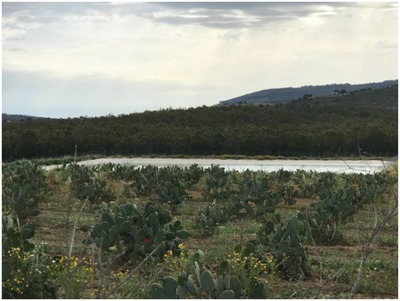
Water pond serving part of the irrigation needs of an *Opuntia ficus‐indica* plantation in Sicily, Italy. (Photograph taken by Mario Pagliaro).

The irrigation water system makes use of two hydraulic electro‐pumps pumping water from two distant wells. The well water is then brought in one case to an artificial pond (**Figure** **4**), and in another to cement reservoirs (Figure 3).

Both the small lake and the water tanks are located in the highest *plateau* of the plantation. Due to the steep slope and large height difference, the water flowing from both reservoirs reaches a 13 atm pressure which is almost five times larger than the 3 atm constant pressure required by the drip irrigation system used throughout the plantation.

Prior to the introduction of the Internet of Things technologies mentioned above, a worker employing up to 40% of the workday slowly opened the valves of the 13 plantation distribution nodes. Besides undertaking continuous corrections lasting 30–40 min per each node, a long time was also required to reach out each subsequent node within the plantation and come back. Finally, when several hours later irrigation was complete, the worker closed all the valves crossing again almost the whole plantation.

**Figure 5:**
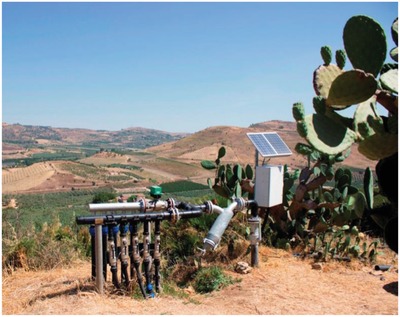
One of the 13 solar‐powered sensor and actuator systems controlling solar‐powered irrigation in a 100 ha *Opuntia ficus‐indica* plantation in Sicily, Italy. (Image courtesy of Agrowireless).

Now, the whole system is completely automated (**Figure** **5**). The pressure within the water networks is monitored by pressure sensors whereas PV electricity‐powered valves and pressure transducers are managed by the wireless control modules only. Once the opening command is received, each module gradually opens the valve controlling the pressure through the pressure transducer.

Each module monitors the pressure every 0.5 s and acts according to plantation manager's will. If, for example, a hole causes a sudden decrease of pressure in the waterworks, the system immediately closes the valve on top notifying the manager via a message sent to the smartphone.

For irrigation, the farming company now uses PV electricity only. In detail, the cement water tanks use an electro‐pump absorbing 18 kW of power. Prior to wireless automation, a worker checked the water level in the tanks and opened the filling valve. The same worker then entered the technical room to check the production of the 30 kW solar PV plant supplying electricity to the plantation during the day, switching on the pump *only* if the power generated by the PV array exceeded 18 kW.

In the late afternoon, the same worker checked again the actual PV production. When it was found insufficient (<18 kW), the worker closed the filling valve and switched off the electro‐pump. Today, an ultrasound level sensor using no mechanical parts suffering from degradation, continuously monitors the water level in the tanks. Another sensor detects the actual power production of the PV array. Both sensors and electro‐pump are now connected to the wireless control module.

When the moisture level in soil diminishes below a threshold chosen by the plantation manager, and only if the power from the PV array exceeds the 18 kW value required by the pump, the system opens the filling valve and switches on the pump.

Filling stops only when tanks are full or when power from the PV array is not enough to meet the pump's power need. In other words, irrigation starts automatically each time the system's software (Agrocontrol) receives information from the ground sensors that the humidity in the ground is too low, and stops when the same sensor informs the system that the humidity threshold preselected has been achieved.

In this way, the plantation company not only optimizes the use of clean solar energy, maximizing self‐sufficiency, but also saves considerable amounts of water through state of the art drip irrigation. The *Opuntia* plant, indeed, requires only water sufficient to wet the plant radical system. All water exceeding the depth of the roots is rapidly wasted through the ground, eventually wasting large amounts of water.

Real‐world data after the first season indicate a reduction in the amount of irrigation water used exceeding 40% compared to the previous irrigation mode.

Finally, worktime previously dedicated to low value‐added activities is freed for more valued tasks. The system, during more than 2 years of continuous operation, has been shown to be highly reliable, with no maintenance interventions.

### 3.2 A Self‐Sufficient Plantation in Australia

**Figure 6:**
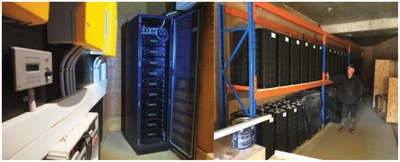
The 48 kWh battery system (left) and the 160 kWh saltwater battery (right) used in an off‐grid avocado plantation in Pemberton, Australia. (Image courtesy of Unlimited Energy Australia).

In Australia's Pemberton, a 5 ha plantation of avocados is now an energy‐independent company using self‐generated renewable electricity only. In 2016, the company's management decided to go completely off‐grid. As of June 21, 2018, the property had been operating for 660 days on self‐generated clean energy generated by a 53 kW PV array. The electricity is stored in a 208 kWh battery storage bank comprised of an older 160 kWh sodium ion battery system and a 48 kWh lithium‐ion battery package (**Figure** **6**).^21^

Likewise to Sicily's *Opuntia* plantation, the avocado plants are irrigated using hydraulic water pumps from underground water, which refills during the rainy winter season. Solar energy powers also the living quarters of the farm's workers.

The digital control technology here is an innovative battery optimizer electronic technology to optimize the performance of Li‐ion cells in a stack, similarly to what happens with electronic devices used to optimize the performance of single PV modules in a string.

In brief, the system assesses the charge of individual cells and the individual battery modules in relation to one another. Exactly as it happens with PV solar cells, the cells in a Li‐ion battery system age differently depending on a variety of factors eventually leading to differences in capacity and internal resistance, and thus between the voltage of each individual cell.^13^

The new bidirectional cell monitoring and balancing system installed in the 48 kWh battery pack in Australia is able to detect and correct such differences on a continuous basis. Excess charges are transferred to any other cell with lower charge in the stack. This bidirectional method of cell balancing reaches a levelized state of charge in a very short time and with minimal energy loss (as heat), with an overall energy conversion efficiency of 92%.

In detail, cells in Li‐ion battery modules age differently leading to different state of charge for modules in the same stack. The active bidirectional balancing method quickly achieves a levelized state of charge with minimal energy loss. A digital optimizer monitors the temperature, voltage, and state of health and of charge of each individual cell or module and controls them within the battery system.

Significantly increasing the service life of individual cells and therefore of the battery, this approach prevents unnecessary heating of the system taking place with batteries using passive balancing in which cells with excess voltage are discharged using fixed or selectable bypass resistances, which in turn would require a cooling system.^22^

Finally, when the battery pack is fully charged, any excess energy coming from the PV array is transferred to a heating element to provide hot water, whereas if the battery charge falls below 60%, appliances are progressively switched off starting from the heating system for the living quarters and ending with the luminaires.

### 3.3 Self‐Sufficient Homes

People living and working in buildings need electricity and heat. Heat requirements, especially in northern countries, are particularly demanding and have led, for instance, the European Union to draft demanding legislation for energy efficiency in buildings.

Hydrogen fuel cells, therefore, are particularly well suited to serve today's and tomorrow's building energy needs since they generate heat along with plentiful electricity.

The uniquely high energy density of compressed hydrogen coupled to over‐abundance of solar radiation in summer months today makes it possible to render energetically self‐sufficient homes even in Sweden. Here, a municipal housing company started in early 2018 a program to progressively make self‐sufficient its homes. As houses undergo renovation, they are also equipped with new hydrogen‐fuelled fuel cell systems, whereas hydrogen produced via water electrolysis is stored in a building separated from the residential buildings.

**Figure 7:**
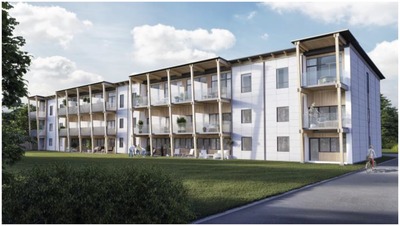
Rendering of the three‐story homes currently under renovation to become off‐grid houses running on PV electricity and solar hydrogen only in Vårgårda, Sweden (Image courtesy of Powercell Sweden, 2018).

The first six homes with 29 apartments each currently undergoing renovation by municipal housing company Vårgårda Bostäder have been designed to go off‐grid to be powered by clean energy only.^23^ The first 29 homes are currently being expanded with a third floor (**Figure** **7**) to accommodate more apartments and new roofs optimized for the use of solar PV modules.

The electricity surplus that is not used during summer is converted into hydrogen with a water electrolyzer equipped with a compressor and stored in pressurized tanks. During winter, at night and in cloudy days when insolation is absent or simply insufficient to provide enough power, hydrogen is combined with air's oxygen in the fuel cells to generate both electricity and heat.

The world's first complex of four family homes fully supported by solar power from photovoltaic modules with excess energy stored in batteries and as solar hydrogen (the Phi Suea House) was inaugurated in Thailand's Chiang Mai in 2015.^24^ Here, solar PV modules with an overall 101.1 kW nominal power generate roughly 14 000 kWh per year. Part of this electricity is converted into compressed H_2_ using an alkaline water electrolyzer. Eventually, 7.5 kg of hydrogen is stored under pressure fuelling a 4 kW fuel cell which, in its turn, keeps the batteries in their ideal state of charge.

Contrary to what is reported in most scholarly publications introducing new electrocatalytic materials, the commercial production of hydrogen via water electrolysis does not use expensive Ir or Pt electrodes, but rather low‐cost Ni‐based electrodes.^9^ What historically limited the industrial production of H_2_ from H_2_O has been the high cost of power which made the cost of water‐derived hydrogen significantly higher when compared to H_2_ obtained via methane reforming in the petrochemical industry.

Now that plentiful and often abundant PV and wind electricity has become available at very low cost, this situation has been reversed making the distributed production of solar hydrogen from water largely convenient.^25^

**Figure 8:**
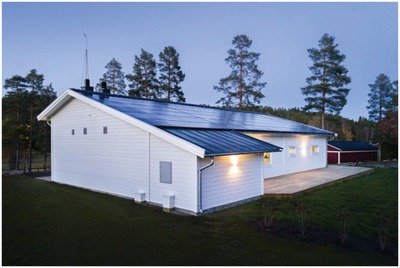
Off‐grid Skellefteå Kraft Zero Sun home in Skellefteå, Swedish Lapland. (Image courtesy of Skellefteå Kraft, picture of Cliff Karlsson).

Even in much less sunny northern Sweden, a self‐sufficient home (**Figure** **8**) uses PV electricity supplied by the PV modules mounted on 122 m^2^ of the roof's surface to meet all the energy (electricity and heat) needs of its residents thanks to a fuel cell powered by solar hydrogen from water electrolysis, and a 144 kWh battery pack.^26^

Disconnected from the grid and from the natural gas network, the “Zero‐Sun” household is a modern one‐story house from the standard range of a Swedish construction company whose only customization is a small upgrade of the window energy class and the presence of an equipment room hosting the battery pack, the water electrolyzer and the H_2_ fuel cell. When batteries are 85% charged, power from the solar PVs is redirected to hydrogen production by water electrolysis. The hydrogen gas is compressed and stored in safe steel tanks from where it is retrieved by a proton exchange membrane (PEM) fuel cell in the evening and night hours, and during winter (November through February, when PV production in Sweden is negligible), to generate electricity and hot water.

We remind that an H_2_ fuel always generates heat along electricity so that by cooling the fuel cell by water, it generates hot water, which is brought back into the house to be used for domestic heating and as sanitary hot water.

When the battery state of charge level goes below a certain threshold, electricity from the fuel cell is used to recharge the battery. The whole process regulating the interaction among solar cells, batteries, electrolyzer and fuel cells is self‐managed via a microcontroller.

In brief, the high energy density of compressed hydrogen, coupled with abundant solar radiation in summer months, makes it possible to cover the need for heat and electricity during the cold and dark winter months even in Swedish Lapland, whereas the PEM fuel cell compact stack^27^ of today generates electricity and hot water in silence and in the highly reliable fashion needed to act as energy generator for buildings and households.

## 4 Perspective and Conclusions

The reported success stories from distant countries have shown how today's digital control technology, coupled with efficient energy storage in Li‐ion battery and solar hydrogen, starts to be actually used by companies and families to access clean, reliable, and cheap solar electricity for all energy end uses, on an annual basis. The management of all the energy (electricity and heat) flows using today's digital control adaptive and self‐learning digital technologies enables us to maximize clean energy generation, reduce losses, and meet the highly variable energy demand smoothly.

One might therefore ask how the solutions identified could scale up, especially in large cities where self‐generation of electricity via building‐integrated PV may cover about 30% of the annual demand with solar electricity (and about 66% of the electricity consumption during daylight hours).^28^

Nardelli and co‐workers have lately discussed how self‐generation coupled with energy storage and digital energy management will actually scale up if structural changes in the liberalized electricity market composed of big generators, retailers, and ever increasing distributed generation with new small generators from renewable energy sources connecting to the grid happen accordingly.^29^ For example, new governance models like energy communities or peer‐to‐peer local markets will need to be deployed as electricity flowing through the grid evolves from being a commodity to be sold in the profit‐driven market, to a commons shared via specific governance models focusing on use.

As the transition to the energy Internet slowly but inevitably takes place across countries, a significant fraction of existing households and organizations self‐produce and self‐manage energy self‐generated via building‐integrated PV, storing the excess produced during the sunny months of the year in solar hydrogen obtained via water electrolysis and Li‐ion batteries. The hydrogen fuel cell, a rapidly emerging energy technology, becomes the other new key technology enabling the transition to energy self‐sufficiency.

Organizations aiming at energy self‐sufficiency based on the aforementioned technologies need energy managers able to articulate a concrete energy transition strategy and deploy it providing guidance to the organization.

For this to happen, managers and entrepreneurs need to expand their literacy to encompass a true understanding of energy, in particular, today's solar energy technologies.^30^ Otherwise, when hearing the word “hydrogen” managers will continue to associate it with the “Hindeburg” fire, the blaze that destroyed a giant fabric balloon coated with highly flammable paint, and not today's hydrogen energy technology in which solar H_2_ is safely stored at high pressure (300–700 bar) in composite material reservoirs capable of resisting without exploding in demanding car crash tests used to evaluate safety of today's fuel cell electric cars.^[9,25]^

At the same time, education in energy management needs to be reshaped, removing the barrier between management and scientific education.^31^

“Leaders in higher education and renewable energy,” has lately concluded a prominent solar energy entrepreneur in the US, “have an obligation to students and citizens of the world to partner and educate the generation of tomorrow on just how viable renewable energy is.”^32^

## Conflict of Interest

The authors declare no conflict of interest.
